# Neuroprotective Effects of Nano-Curcumin against Cypermethrin Associated Oxidative Stress and Up-Regulation of Apoptotic and Inflammatory Gene Expression in Rat Brains

**DOI:** 10.3390/antiox12030644

**Published:** 2023-03-04

**Authors:** Mohammad Ashafaq, Sohail Hussain, Saeed Alshahrani, Rahimullah Siddiqui, Mohammad Intakhab Alam, Manal Mohamed Elhassan Taha, Yosif Almoshari, Saad S. Alqahtani, Abdulmajeed M. Jali, Hashim M. Aljohani

**Affiliations:** 1 Department of Pharmacology and Toxicology, College of Pharmacy, Jazan University, Jazan 45142, Saudi Arabia; 2 Department of Pharmaceutics, College of Pharmacy, Jazan University, Jazan 45142, Saudi Arabia; 3 Substance Abuse Research Center (SARC), College of Pharmacy, Jazan University, Jazan 45142, Saudi Arabia; 4 Department of Clinical Pharmacy, College of Pharmacy, King Khalid University, Abha 62529, Saudi Arabia; 5 Department of Clinical Laboratory Sciences, Collage of Applied Medical Sciences, Taibah University, Madinah 41491, Saudi Arabia; 6 Department of Pathology and Laboratory Medicine, Collage of Medicine, University of Cincinnati, Cincinnati, OH 45221, USA

**Keywords:** cypermethrin, nano-curcumin, antioxidants, oxidative stress, inflammation, apoptosis, neurotoxicity

## Abstract

Cypermethrin (CPM) is the most toxic synthetic pyrethroid that has established neurotoxicity through oxidative stress and neurochemical agitation in experimental rats. The toxic effects are supposed to be mediated by modifying the sodium channels, reducing Na-K ATPase, acetylcholine esterase (AchE), and monoamine oxidase (MAO). The use of curcumin nanoparticles (NC) that have potent antioxidant, anti-inflammatory and antiapoptotic properties with improved bioavailability attenuates neurotoxicity in rat brains. To test this hypothesis, animals were divided into five groups, each having six animals. Group-I control received vehicle only, while Group-II was treated with 50 mg/kg CPM. Group-III and Group-IV received both CPM and NC 2.5 mg/kg and 5 mg/kg, respectively. Group-V received 5 mg of NC alone. The CPM and NC were given by oral route. Afterwards, brain antioxidant status was measured by assessing lipid peroxidation (LPO), 4-HNE, glutathione reduced (GSH), antioxidant enzyme catalase, and superoxide dismutase (SOD) along with neurotoxicity markers Na-K ATPase, AchE, and MAO. Inflammation and apoptosis indices were estimated by ELISA, qRT-PCR, and immunohistochemistry, while morphologic changes were examined by histopathology. Observations from the study confirmed CPM-induced neurotoxicity by altering Na-K ATPase, AchE, and MAO, and by decreasing the activity of antioxidant enzymes and GSH. Oxidative stress marker LPO and the level of inflammatory interleukins IL-6, IL-1β, and TNF-α were notably high, and elevated expressions of Bax, NF-kB, and caspase-3 and -9 were reported in CPM group. However, NC treatment against CPM offers protection by improving antioxidant status and lowering LPO, inflammation, and apoptosis. The neurotoxicity marker’s enzyme successfully attenuated after NC treatment. Therefore, this study supports the administration of NC effectively ameliorated CPM-induced neurotoxicity in experimental rats.

## 1. Introduction

Cypermethrin (CPM) is a class-II synthetic pyrethroid, traditionally applied to manage insects at a commercial scale in agriculture, domestic practice, and in medicine for topical applications [1]. It has the ability to cross the blood-brain barrier and facilitate central nervous system (CNS) function by modulating sodium channels in the brain [2]. This further induces the development of neurotoxicity by encouraging oxidative stress, inflammation, and apoptosis in the brain [3,4]. As a result, CPM holds a fast-acting neurotoxin action. Despite their widespread use, there is growing interest in their neurotoxic effects on humans. Through extensive research, it has been accepted that CPM exposure to humans results in neurotoxicity and neurodegenerative diseases [5,6]. Insecticides can interrupt the function of vital organs in the body and bring about endocrine, cardiovascular, reproductive, renal, and nervous system disorders [7].

CPM undergoes metabolic degradation by the liver Cytochrome-P450 enzyme, which generates reactive oxygen species (ROS), leading to oxidative stress in experimental animals [8]. The brain is highly sensitive to ROS-induced oxidative stress because of its high content of polyunsaturated fatty acids and poor cellular defenses [9]. Additionally, CPM-mediated oxidative stress may have depleting effects on antioxidant enzymes, such as catalase, superoxide dismutase (SOD), and glutathione (GSH), hence reducing antioxidant defenses [10]. 

CPM also exerts neurotoxic consequences by altering voltage-dependent sodium channels and integral protein ATPase, the activity of acetylcholine esterase (AchE), and monoamine oxidase enzyme in the neuronal membrane [11,12]. CPM exhibits neurotoxicity by causing excitation of the central nervous system by modulating ion channels in the CNS, which includes the opening of sodium channels, leading to hypo-polarization and hyper-excitation of the neurons [13]. In addition to oxidative stress, activation of the inflammatory and apoptotic pathways is also implicated in CPM-mediated neurotoxicity [14]. Neuronal injury may be facilitated by the activation of microglia, which upregulate inflammatory cytokines such as tumor necrosis factor-α (TNF-α), interleukin-1β, and IL-6, which may increase neurotoxicity [15]. ROS-mediated DNA damage and cell death encourage apoptosis in CPM-exposed rat brains [16]. 

Accumulating evidence indicates that oxidative stress plays a major role in CPM-induced neurotoxicity, which attracts the potential use of pharmacologically active antioxidants. Medicinal plant products are known to exert their protective effects by scavenging free radicals and improving antioxidant levels. Curcumin, derived from the rhizomes of *Curcuma long* turmeric, is used as a spice in cooking [17]. Curcumin exhibits potent antioxidant, anti-inflammatory, and antiapoptotic activity [18,19,20]. Curcumin is a potent ROS inhibitor and has shown neuroprotective properties [21]. Naturally occurring antioxidants show less bioavailability, which is why a pharmaceutical application is partial. Through research studies, it has been well established that bioavailability can be improved by nano-formulations, so its pharmaceutical efficacy in neurotoxicity may be enhanced [22]. Nano-formulation can also decrease the dose and potentiate the efficacy of antioxidant compounds. The aim of the study is to determine the primary mechanisms involved in CPM-induced neurotoxicity and its treatments by the nano-formulation of curcumin. 

## 2. Materials and Methods

### 2.1. Chemicals

Commercially available curcumin, cypermethrin, and another reagent of the highest analytical grade were sourced from Sigma Chemicals, Balcatta, WA, USA. Assay kits of IL-6, IL-β, TNF-α, caspases-3,9, and all antibodies for immunohistochemistry were purchased from Abcam (Cambridge, UK) Sigma. RNA isolation and cDNA kits for qRT-PCR were procured from Bio-Rad and Applied Biosystems, while primers were from Macrogen Inc., Seoul, Republic of Korea.

### 2.2. Experimental Animals

Male Wistar rats weighting 200–230 g were acquired from the Medical Research Center at Jazan University. The animals were kept for adaptation before the commencement of the experiments in the College of Pharmacy in standard lab environments (temperature 25 ± 2 °C) with 12 h light–dark cycles. Animals had free access to ordinary pellet diets and water. The experimental procedures complied with the ethical regulations by the Institutional Research Review and Ethical Committee (IRREC), Jazan University, and studies were completed in accordance with the standard Guidelines of the Ethical Committee (Animal Ethical Approval Number: 902/207/1443).

### 2.3. Preparation of Nano-Curcumin, Particle Size, Polydispersity Index and Zeta Potential

Nanostructured lipid carriers (NLC) containing curcumin (nano-curcumin) were prepared using the hot homogenization method, as reported earlier [23]. Briefly, the molten solid lipid (stearic acid) was mixed with liquid lipid and curcumin was added and mixed. The aqueous phase containing surfactants was heated. Both the phases (lipid and aqueous) were homogenized using a homogenizer for 20 min at a speed of 6000 rpm. It was then filtered through Whatman filter paper and stored for different studies. The size of the nanoparticle and PDI (polydispersity index) were measured by photon correlation spectroscopy using Malvern Zetasizer (Zetasizer Nano ZS, Worcestershire, UK). The surface charges of nanoparticles called zeta potential were measured by using a Malvern Zetasizer. The morphology of the NC was determined using transmission electron microscopy (JEM1010, JEOL, Tokyo, Japan). 

### 2.4. Study Design

Animals were randomly allocated into five groups, and each group was assigned six rats. Group-I served as control and only received the vehicle for 15 days, while Group-II received CPM only 50 mg/kg for 15 days. The dose of CPM was selected on the basis of a literature study reported earlier [24,25]. Group-III and Group-IV were subjected to CPM as with Group-II along with NC 2.5 mg/kg and 5 mg/kg for 10 days, commencing 5 days after CPM treatment. Group-V were subjected to NC 5 mg/kg only. Every group received all the treatment orally. 

### 2.5. Sample Preparation for Oxidative Stress and ELISA Assay

Experimentation was terminated by sacrificing animals of every group, and the frontal cortex of the brain was procured quickly for homogenate, PMS, and ELISA assays. Oxidative stress and neurotoxicity were determined by assaying enzyme and non-enzyme parameters in homogenate and PMS (5% *w*/*v*, 10 mM Tris–HCl, pH 7.4), as described earlier [22].

### 2.6. Determination of Oxidative Stress

The brain homogenate and PMS were used to estimate the content of lipid peroxidation in terms of TBARS, GSH, activities of SOD, and Catalase, as described earlier [22,26,27]. For the TBARS assay, 0.25 mL homogenate were incubated at 37 °C and 0 °C. After 1 hr., 0.5 mL of 5% trichloroacetic acid and 0.5 mL of 0.67% thiobarbituric acid was added to both sets of samples. The sample tube was centrifuged at 3000g for 15 min. and supernatant was positioned in a boiling water bath for 10 min. The absorbance was recorded at 535 nm. GSH was examined by incubating the sample in sulphosalicylic acid for 1 hr. at 4 °C. The sample tube contained 0.1 mL of a supernatant after centrifugation, 1.7 mL PB (0.1 M, pH 7.4), and 0.2 mL DTNB. The sample absorbance was recorded immediately after adding DTNB. SOD activity was estimated by observing the autooxidation of (−)-epinephrine at pH 10.4 for 3 min at 480 nm. The sample tube contained 0.2 mL brain tissue PMS and 50 mM (pH, 10.4) glycine buffer. The reaction was initiated after adding of (−)-epinephrine, and enzyme activity was calculated by using molar extinction coefficient of 4.02 × 103 M^−1^ cm^−1^. The SOD activity was expressed as nmol (−)-epinephrine protected from oxidation/min/mg protein. Catalase assay contains 0.05 M PB, 0.05 mL PMS and 0.019 M hydrogen peroxide. The sample absorbance was observed at 240 nm. Catalase activity expressed as nmol of H_2_O_2_ consumed/min/mg protein. The protein content of the samples was explored by the scheme of Lowry et al. [28].

### 2.7. Determination of Neurotoxicity Markers

Neurotoxicity in brain tissue was explored by measuring Na/K-ATPase, AchE, and MAO activity, as explored previously [29]. 

### 2.8. Assay of Inflammatory Cytokine (IL-6, IL-1β and TNF-α) and Apoptosis Markers (Caspase-3 and -9)

The manufacturer method was adopted to assay IL-6, IL-1β, TNF-α, and caspase-3 and -9 in brain homogenates using ELISA kits (Abcam, Cambridge, UK). The 96 well plate was read at the specific wave length mentioned in the manufacturer method by Microplate Reader (ELx 800TM BioTek, Winooski, VT, USA).

### 2.9. RNA Isolation and cDNA Preparation 

Total RNA from control, CPM, and NC treated groups was isolated from the brain cortex using the Aurum™ Total RNA Mini Kit (Cat#7326820, *BIO-RAD*). cDNA was synthesized using a High-Capacity cDNA Reverse Transcription Kit with RNase Inhibitor (Appliedbiosystems A25918). Quantification of RNA and cDNA was performed by taking the absorbance at 260 nm. The resulting cDNAs were used as templates for qRT-PCR in the CFX96 Real-Time PCR System (*BIO-RAD*) using PowerUp™ SYBR green master mix (Appliedbiosystems A25742). The primers used for qRT-PCR and gene accession no. are mentioned in Table 1, and reaction conditions were adopted as given in the kit. Specific primer sequences of Bax, NF-kB, TNF-α, and caspase-3 were obtained from Macrogen Inc. (Seoul, Republic of Korea). The value of each sample was normalized to β-actin expression as a reference standard, while gene expression presented as relative expressions was evaluated as the 2^^ΔΔ^ CT method [30].

### 2.10. Immunohistochemical Staining

Rats were perfused transcardially with cold phosphate buffer saline (PBS; 0.1 M, pH 7.2) and followed by formaldehyde, glacial acetic acid, and methanol (1:1:8, *v*/*v*), as described by Khan et al. [9] The brains were removed quickly, and post-fixed in the same fixative for 24 h. After 24 h, they were transferred into 30% sucrose in 0.1M PB until they sank. The fixed tissues were embedded in the optimal cutting temperature (OCT) and frozen at −20 °C. Coronal sections of frontal cortex of 10-μm thicknesses were cut on a cryostat and collected on gelatin-coated slides. Sections were treated with blocking solution for endogenous peroxidase activity (3% H_2_O_2_) and washed with PBS. After that, sections were incubated overnight in a humid chamber at 4 °C with anti-4 HNE, anti-Bax, and an anti-NF-kB antibody of a dilution of 1:100. The unbound antibody was removed by washing, and it was incubated with biotinylated, a secondary antibody dilution of 1:5000, for 2–4 h at room temperature in the humid chamber. Diaminobenzidine (DAB) was applied on sections to develop color for microscopic observation. The sections were dehydrated by ethanol and xylene and mounting media to cover slipped. The Immunohistochemical stain was recorded by a light microscope at 40× magnification.

### 2.11. DNA Isolation and Fragmentation Assay

Genomic DNA was isolated from 50 mg of brain tissue and homogenized in a digestion buffer, as described by the manufacturer (QIAamp DNA–QIAGEN). The isolated DNA samples were further quantified by Spectrophotometers, as described earlier [23]. To examine DNA fragmentation, 15 µg samples from each group were loaded onto 1.5% agarose gel, holding 0.5 µg/mL of ethidium bromide. The DNA sample was electrophoresed at 80 V for 20 min for the analysis of the fragmentation, which was detected with a UV transilluminator.

### 2.12. Brain Histology

Perfused brain tissue was isolated from all group animals, cleaned using physiological saline (0.89%), and kept in 10% formaldehyde for further sectioning. Fixed tissue was embedded in beeswax paraffin and tissue blocks were prepared. The 5 μm thick coronal sections of the frontal cortex were collected from paraffin-embedded tissues. Further sections were processed through deparaffinization with the exchange of xylene, ethanol, and water. The deparaffinized sections stain in hematoxylin and eosin, and sections were mounted to cover slipped. Images were captured using a light microscope.

### 2.13. Statistical Study

GraphPad Prism8 was used to scrutinize the data obtained from each group by applying a one-way analysis of variance (ANOVA) with the post hoc Tukey test. The data of the measured parameters are presented as mean ± SE, with a *p*-value less than 0.05 considered statistically significant. 

## 3. Results

### 3.1. Particle Size, Polydispersity Index and Zeta Potential 

NC was prepared and characterized successfully by the methods reported earlier by our group [23]. The average particle size was observed to be 60.58 ± 4.16 d.nm (*n* = 3), with a polydispersity index (PDI) of 0.252 ± 0.019 (*n* = 3). The zeta potential of NC was found to be −48.57 ± 2.61 mV.

### 3.2. Effect of NC and CPM Treatment on Oxidative Stress Markers

Major markers of oxidative stress and antioxidant enzyme activity were determined by a spectrophotometric method in brain tissue. The raised content of TBARS and declined GSH, SOD, and catalase were explored in the CPM-treated group, as compared to the control (*p* < 0.01; *p* < 0.001). NC treatment significantly cut down the increased content of TBARS (*p* < 0.05; *p* < 0.01), whereas it protected the level of GSH, SOD, and catalase activity (*p* < 0.05; *p* < 0.01 and *p* < 0.001) in the CPM-treated NC group (CPM+NC). However, NC alone does not show any significant changes when examined with the control group (Table 2).

### 3.3. Effect of NC on CPM-Induced Neurotoxicity

**Figure 1:**
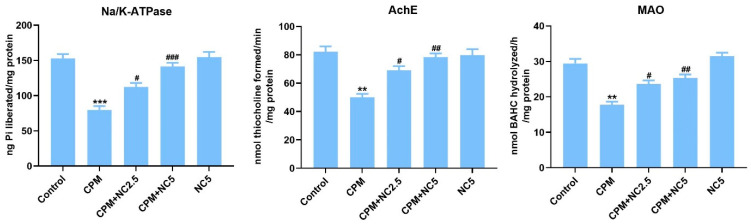
Effect of NC administration on neurotoxicity biomarkers (Na/K ATPase, AchE and MAO) against CPM-induced neurotoxicity. Data stated as mean ± SE (n = 6). ** *p* < 0.01; *** *p* < 0.01 vs. control, # *p* < 0.05; ## *p* < 0.01; ### *p* < 0.001 vs. CPM group.

Reduced levels of Na-K-ATPase, AchE, and MAO (Figure 1) were observed in CPM-induced neurotoxicity when examined with the control group (*p* < 0.01; *p* < 0.001). Neurotoxicity markers were significantly recovered in NC treatment compared to the CPM group due to its potent antioxidant and neuroprotective activity (*p* < 0.05; *p* < 0.01; *p* < 0.001).

### 3.4. Role of NC on CPM-Induced Proinflammatory Cytokines

**Figure 2:**
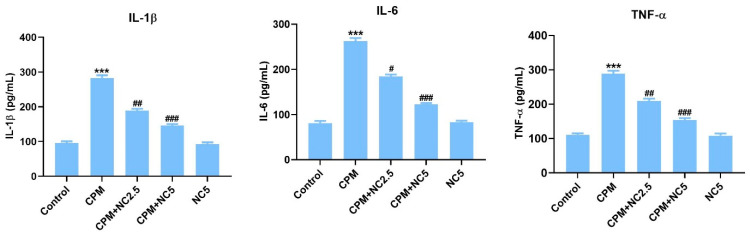
Effect of NC treatment on IL-1β, IL-6, and TNF-α against CPM-induced neurotoxicity. Value shown as mean ± SE (n = 6). *** *p* < 0.001 vs. control, # *p* < 0.05; ## *p* < 0.01; ### *p* < 0.001 vs. CPM group.

Oral treatment of CPM in rats activates proinflammatory cytokines IL-6, IL-1β, and TNF-α compared to the control group rats (*p* < 0.001), while NC treatment successfully reduces these markers in brain tissue (*p* < 0.05; *p* < 0.01; *p* < 0.001) (Figure 2).

### 3.5. Role of NC on CPM-Induced Apoptosis

**Figure 3:**
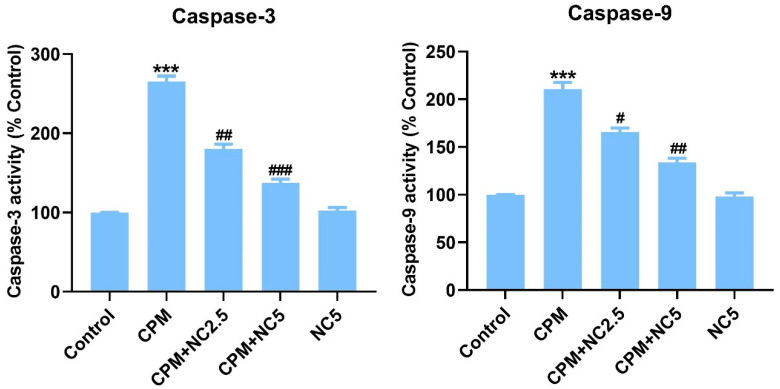
Shows successful suppression of caspase-3 and -9 by NC treatment. Statistical value expressed as mean ± SE (n = 6). *** *p* < 0.001 vs. control, # *p* < 0.05; ## *p* < 0.01; ### *p* < 0.001 vs. CPM group.

Colorimetric assay of caspase-3 and -9 displayed activation of these apoptotic markers in the CPM-treated group when equated to the control (*p* < 0.001), but NC treatment significantly suppresses the activation of caspase-3 and -9 dose dependently (*p* < 0.05; *p* < 0.01; *p* < 0.001) (Figure 3).

### 3.6. Role of NC on RNA Expression Levels

**Figure 4:**
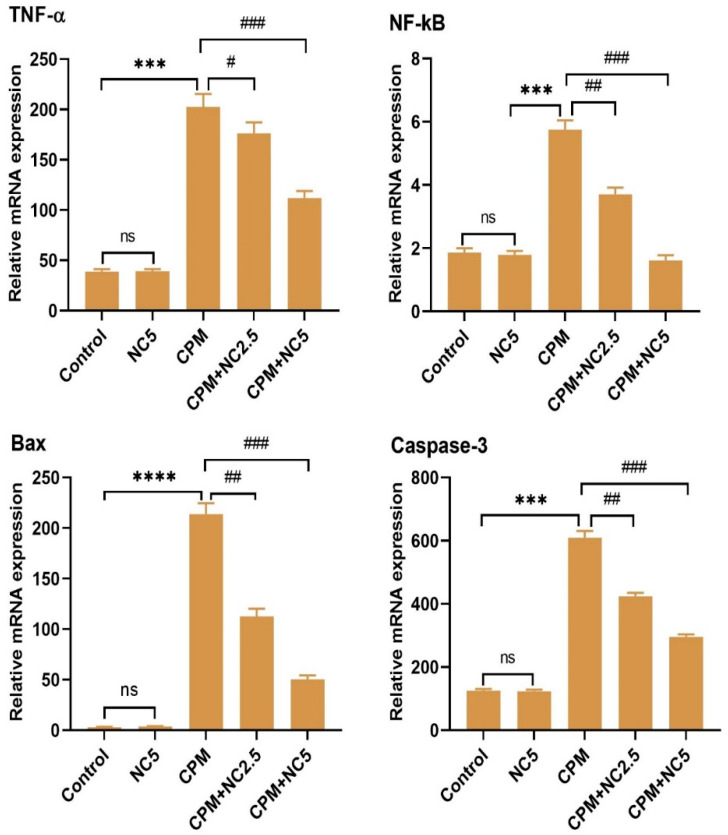
Representation of effect of NC against CPM treatment on mRNA transcription level of TNF-α, NF-kB, Bax and caspase 3. Values signify the mean ± SE (n = 3) of samples. **** *p* < 0.0001; *** *p* < 0.001 vs. control, # *p* < 0.05; ## *p* < 0.01; ### *p* < 0.001 vs. CPM, ns = non-significant.

The mRNA expression level of inflammatory and apoptotic indices was confirmed by qRT-PCR, as shown in Figure 4. On observation of the tested results, we see that the CPM group significantly amplified the expression level of TNF-α, NF-kB, BAX, and caspase-3 as equated to the control group (*p* < 0.001). However, NC administration significantly reduces the expression level of inflammatory and apoptotic responses dose dependently when compared to the CPM group *(p* < 0.05; *p* < 0.01; *p* < 0.001). Only NC did not show any effects after comparing it with the control.

### 3.7. NC Suppresses CPM-Induced 4-HNE, Bax, and NF-kB Expression 

**Figure 5:**
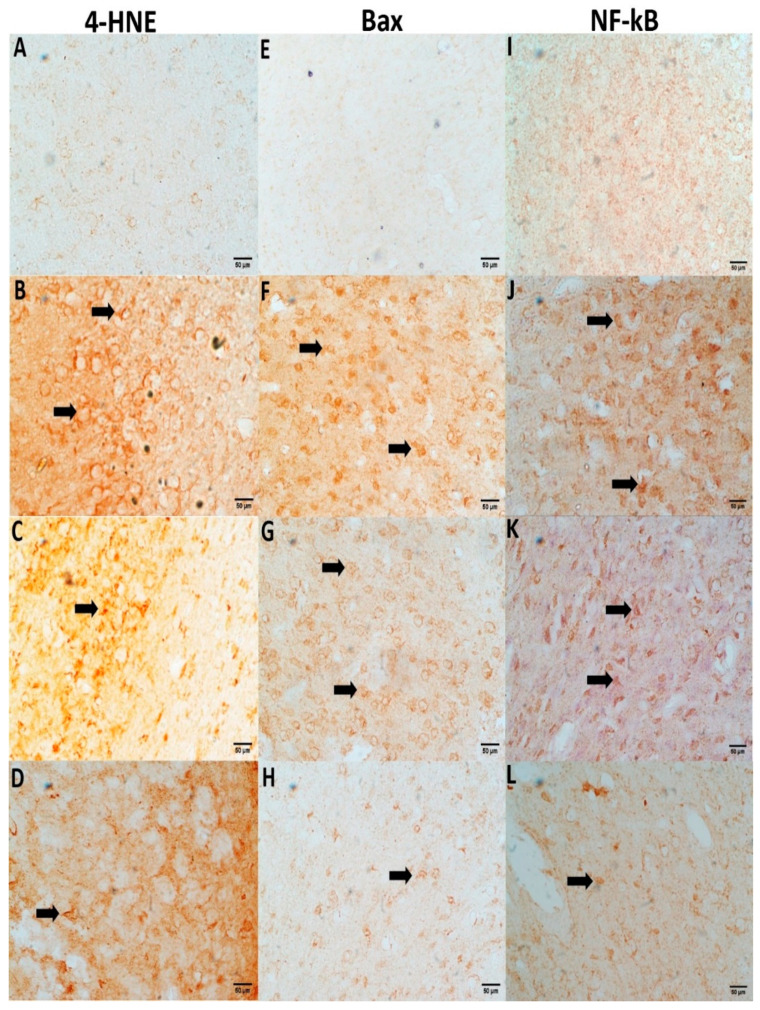
NC treatment suppresses immunochemical staining for 4-HNE, Bax, and NF-kB. Slides from the control group (**A**,**E**,**I**) show negative stains of neurons. A positive neuronal staining with high density was observed in the CPM group for 4-HNE (**B**), Bax (**F**) and NF-kB (**J**) (arrows). However, Positivity in neurons staining for the NC-treated group (2.5 and 5 mg) were successfully reversed as compared to the CMP group (4-HNE (**C**,**D**), Bax (**G**,**H**), and NF-kB (**K**,**L**).

Immunochemical staining confirms a significant elevation in 4-HNE, Bax, and NF-kB positive cells in CPM-treated rat brain tissues. In the section gained from the control group, neurons bare a negative immunoreaction (Figure 5), while fewer immune positive cells were examined in NC-treated groups that offered successful suppression in 4-HNE, Bax, and NF-kB compared to the CPM group. However, no immune staining cells were examined in the NC-alone group (data not shown).

### 3.8. NC Treatment Protect CPM-Induced DNA Fragmentation 

**Figure 6:**
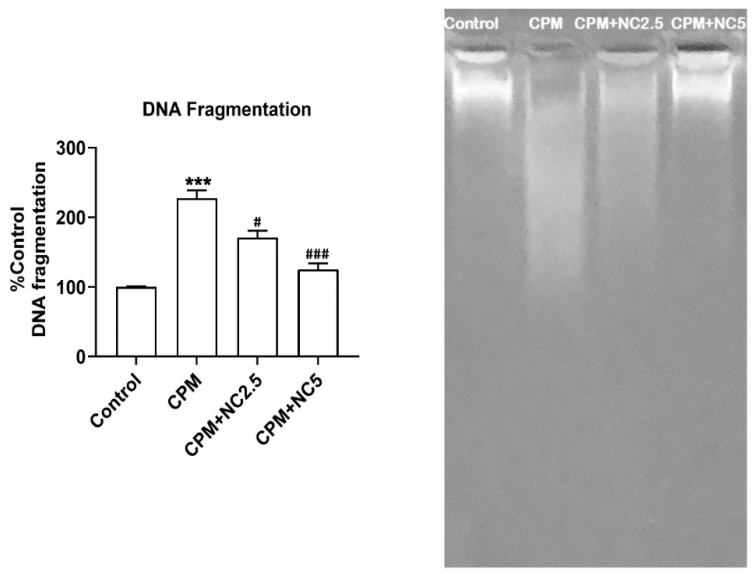
Administration of NC offers excellent protection against CPM-induced DNA fragmentation. Outcomes are presented as the mean ± SE (n = 4) of each group. *** *p* < 0.001 vs. control, # *p* < 0.05; ### *p* < 0.001 vs. CPM. NC shields DNA fragmentation as shown in lane-3 and lane-4 as allied to CPM (lane-2). However, CPM triggers DNA breaks in lane-2 as compared to control lane-1.

CPM-induced DNA damage is a hallmark of apoptotic cell death. The protective role of NC against CPM exposure on DNA integrity is shown in Figure 6. CPM promotes DNA fragmentation in the CPM group (227.13% *p* < 0.001), as likened to the control. NC co-administration (2.5 and 5 mg), meanwhile, significantly shielded DNA fragmentation to 171.09% and 125.11% (*p* < 0.05; *p* < 0.001), individually, as associated to the CPM. Genomic DNA migration is shown in the agarose electrophoresis from the control and treated groups (Figure 6). CPM-induced marked DNA fragmentation shown in the DNA laddering in the CPM-treated group as compared to the control group. Decreased DNA smearing was examined in the CPM+NC (2.5 and 5 mg) group, representing DNA protection. No DNA smearing was observed in NC-alone group.

### 3.9. NC Protects CPM-Induced Histopathological Changes

**Figure 7:**
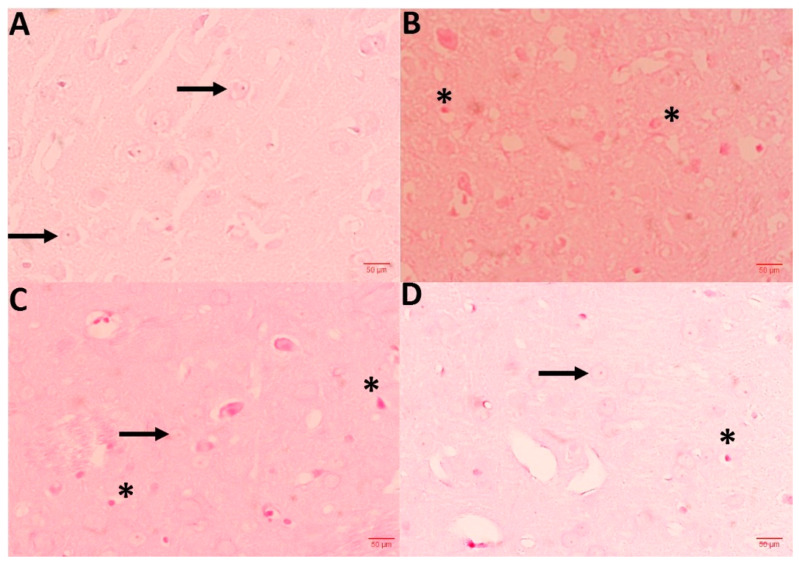
Shows histopathological abrasions of brain sections. (**A**) Control group exhibits normal neuronal histology (arrows), while CPM group (**B**) displays Necrotic cells (star). A dose of NC 2.5 and 5 mg (**C**,**D**) effectively protected neuronal cells against CPM-induced histopathologic alteration. Slide from this section exhibits scarce necrotic cells in comparison with CPM.

To access the CPM-induced neurodegenerative alteration, 5 µm thick brain tissue sections were obtained from all groups and stained in H&E. Sections from the control group represent the normal architecture and morphology of neuronal cells, while the CPM group exhibits markable changes, such as vacuole development, the presence of pycnotic nuclei, and infiltrating cells. Sections from CPM group treated with NC displayed re-establishing protection, numbers of pycnotic nuclei, and the decrease in vacuoles, regaining normal architecture of neuronal cells. NC alone did not represent any morphological alteration when compared with the control group (data not shown) (Figure 7).

## 4. Discussion

The widespread application of CPM has established a significant risk of neurotoxicity involving deteriorated antioxidant status, neurotoxicity marker enzymes, and histopathological changes in brain tissue [31]. Curcumin, with its potent neuroprotective properties [11], has become an attractive alternative treatment tool for various neurological disorders. Due to its poor bioavailability after systemic administration, its neuroprotective activity decreases. This study explored the curcumin incorporated nano-lipid carrier (NC), which has improved bioavailability and enhanced neuroprotective effects in CPM-induced neurotoxicity in rats. A 2-week exposure to 50 mg/kg of CPM exhibited significant impairment in antioxidant indices along with marked neuronal injuries. Remarkably, our findings revealed that NC treatments could efficiently improve antioxidant status and dismiss the pathological changes in the brain. 

Extensive exposure to CPM has been shown to interfere with oxidative processes, which provoke oxidative damage through the development of ROS [10]. This results in a further decline in antioxidant enzyme activity, and it could encourage damage to lipid, protein, and DNA [32]. The extent of oxidative damage is determined by the balance between oxidants and pro-oxidants. TBARS is a major marker of LPO and oxidative stress in the brain [33]. The high content of polyunsaturated fatty acids, comparatively low antioxidant capacity, and scarce cell repair action in the brain are implicated in oxidative damage [34]. Enhanced LPO content, low GSH, moderate SOD, and CAT enzyme activity were explored in the CPM-treated group as compared to the control group. Curcumin is well recognized for its characteristic and potent antioxidative activities. That is why NC administration represents significant progress in the antioxidant enzyme’s GSH content and cuts down LPO levels in the CPM-treated group dose dependently. This finding is comparable with other recently reported studies, where CPM is incorporated into oxidative stress [3,35]. 

CPM seem to be quickly circulated throughout body organs, where they interfere with cellular biochemicals such as enzyme activity, membrane lipid, and nerve communication. [33]. CPM is a well-known pesticide implicated in both the modulation of Na channels [36] and the inhibition of membrane bound integral protein Na/K-ATPase and neurotransmitter metabolizing enzymes AchE and MAO [2]. AchE, prevalent throughout the brain, mediates the breakdown of cholinergic neurotransmitters into choline and acetate, whereas MOA has a pivotal role in stress-related disorders. The findings of the present study showed that CPM-induced neurotoxicity mediated through the inhibition of Na/K-ATPase, AchE, and MAO enzyme. The CPM-treated group showed a deteriorated level of activity of Na/K-ATPase, AchE, and MAO enzymes compared to the control. However, NC administration significantly improved inhibited enzymes in the brain when compared to the CPM group. These findings are in harmony with those of previous studies [2,37].

CPM-induced neurotoxicity is mediated through the emergence of ROS, a decline in antioxidant defense, and the inhibition of neurochemical enzymes. This further prompts inflammatory and apoptotic incidents due to its impoverished cell defense systems, since neuroinflammation and apoptosis have been established in numerous brain disorders, including CPM-induced neurotoxicity [8,38]. Caspase-3 and -9 were upregulated, which are markers of apoptosis in the brain, whereas high levels of IL-6, IL-1β, and TNF-α were found after 15 days of exposure of CPM-induced apoptosis and inflammation. RNA expression of TNF-α, NF-kB, BAX, and caspase-3 was further confirmed by qRT-PCR, and amplified expression of these markers was also confirmed by previously reported studies [39,40]. Neuroinflammation in the brain was modulated by pro-inflammatory regulators evident in CPM-treated group. However, the excellent anti-inflammatory, anti-apoptotic, and neuroprotective properties of NC significantly and dose dependently defended CPM-induced inflammation and apoptosis in the NC-treated group when compared to the CPM-alone group. These findings are also in harmony with other studies where CPM-induced toxicity was significantly attenuated by naturally-derived antioxidants in rats [16,41]. The role of LPO, inflammation, and apoptosis in CPM-induced neurotoxicity was also evident through the immunohistochemical staining of rat brain tissues. 4-HNE is the major marker of lipid damage in terms of the TBARS species in CPM-treated brain tissue. Expressions for 4-HNE positive staining in CPM-treated groups enhanced significantly, whereas NC treatment suppressed expression dose dependently. Furthermore, abundant immunopositive cells for 4-HNE, Bax, and NF-kB were seen in the CPM group compared to the control group. However, NC treatment offered protection in terms of reduced immunopositive cells compared to the CPM group. 

Activation of caspases promotes DNA fragmentation, observed as a laddering pattern in the CPM groups [42]. DNA fragmentation is the hallmark of apoptosis, which was observed as a smear in CPM-treated group. However, NC treatment significantly protected DNA fragmentation against CPM, which is in harmony with other studies reported earlier [16]. Along with DNA damage, histopathological observations also revealed neuronal cell death, which supports our hypothesis of CPM-induced neurotoxicity in the rat brain. The CPM group exhibits neuronal degeneration characterized by the presence of pyknotic nuclei, vacuolization, and infiltrating cells compared to the control group. However, these neuronal alterations were attenuated by NC treatment dose dependently when examined along with the CPM group alone. Our results were in agreement with those of Yadav et al. [43] and Ali et al. [3], who found that CPM treatment occasioned neuronal degeneration, vacuolization, and pycnotic nuclei. 

## 5. Conclusions

In conclusion, the current study demonstrated that CPM exposure caused neurotoxicity, which was associated with oxidative stress, altered neurotoxicity indicators, histopathological changes, increased inflammation, and apoptosis in the rat brain. Co-treatment with NC produces a significant protective impact against CPM exposure. Due to the widespread applications of CPM in domestic, agricultural, and industrial settings, its application should be minimized, and attention should be paid to CPM sources. Furthermore, the protective effects of NC against CMP-induced neurotoxicity may be mediated primarily by its improved bioavailability, as well as antioxidant, anti-inflammatory, and anti-apoptotic activities. 

## Figures and Tables

**Table 1:** qRT-PCR sequences.

Oligo	Primer Sequences	Tm (c)	Gene Accession Number
TNF-α	5′-CCACCACGCTCTTCTGTCTAC-3′ Forward 5′-ACCACCAGTTGGTTGTCTTTG-3′ Reverse	63.3 59.4	NC_051355
NF-kB	5′-CATGAAGAGAAGACACTGACC-3′ Forward 5′-TGGATAGAGGCTAAGTGTAGA-3′ Reverse	59.4 57.4	NM_001415012
Bax	5′-GCCTCCTTTCCTACTTCGGG-3′ Forward 5′-CTTTCCCCGTTCCCCATTCA-3′ Reverse	62.5 60.5	S76511
Caspase-3	5′-GCTACGATCCACCAGCATTT-3′ Forward 5′-ATGCCACCTCTCCTTTCCTT-3′ Reverse	58.4 58.4	NM_012922
Beta actin	5′-CAACCTTCTTGCAGCTCCTC-3′ Forward 5′-TTCTGACCCATACCCACCAT-3′ Reverse	60.5 58.4	AA955227

**Table 2:** Effect of NC treatment on CPM-induced brain stress.

Groups	TBARS (nmol/g Tissue)	GSH (DTNB Conjugate Formed/mg Protein)	SOD (nmol Epinephrine Protected from Oxidation/min/mg Protein)	CAT (nmol of H_2_O_2_ Consumed/min/mg Protein)
Control	1.89 ± 0.26	10.71 ± 1.02	210.61 ± 3.17	8.72 ± 0.14
CPM	3.47 ± 0.91 *** (83.59) ^a^	3.89 ± 0.44 *** (−63.67) ^a^	127.39 ± 2.79 *** (−65.32) ^a^	4.04 ± 0.66 ** (−51.44) ^a^
CPM+NC2.5	2.56 ± 0.21 ^#^ (−26.22) ^b^	5.64 ± 0.98 ^##^ (44.98) ^b^	159.20 ± 2.86 ^#^ (24.97) ^b^	6.33 ± 0.21 ^##^ (56.68) ^b^
CPM+NC5	1.98 ± 0.09 ^##^ (−42.93) ^b^	6.93 ± 0.77 ^###^ (78.14) ^b^	192.05 ± 5.28 ^##^ (50.75) ^b^	7.39 ± 0.47 ^###^ (82.92) ^b^
NC5	1.88 ± 0.32 (−0.52)	11.03 ± 1.26 (2.98)	213.11 ± 4.09 (67.28)	9.12 ± 0.57 (9.61)

Value expressed as mean ± SE (n = 6). ** *p* < 0.01; *** *p* < 0.001 vs. control group. ^#^ *p* < 0.05; ^##^ *p* < 0.01; ^###^ *p* < 0.001 vs. CPM-treated group. ^a^ Values in parentheses indicate the percentage change versus Control. ^b^ Values in parentheses indicate the percentage change versus CPM.

## Data Availability

The data available within the articles.
